# Nuclear Receptor-Like Structure and Interaction of Congenital Heart Disease-Associated Factors GATA4 and NKX2-5

**DOI:** 10.1371/journal.pone.0144145

**Published:** 2015-12-07

**Authors:** Sini Kinnunen, Mika Välimäki, Marja Tölli, Gerd Wohlfahrt, Rami Darwich, Hiba Komati, Mona Nemer, Heikki Ruskoaho

**Affiliations:** 1 Division of Pharmacology and Pharmacotherapy, University of Helsinki, Helsinki, Finland; 2 Institute of Biomedicine, Department of Pharmacology and Toxicology, University of Oulu, Oulu, Finland; 3 Orion Pharma, Computer-Aided Drug Design, Espoo, Finland; 4 Laboratory of Cardiac Development and Differentiation, Department of Biochemistry, Immunology and Microbiology, University of Ottawa, Ottawa, Canada; The University of Queensland, AUSTRALIA

## Abstract

**Aims:**

Transcription factor GATA4 is a dosage sensitive regulator of heart development and alterations in its level or activity lead to congenital heart disease (CHD). GATA4 has also been implicated in cardiac regeneration and repair. GATA4 action involves combinatorial interaction with other cofactors such as NKX2-5, another critical cardiac regulator whose mutations also cause CHD. Despite its critical importance to the heart and its evolutionary conservation across species, the structural basis of the GATA4-NKX2-5 interaction remains incompletely understood.

**Methods and Results:**

A homology model was constructed and used to identify surface amino acids important for the interaction of GATA4 and NKX2-5. These residues were subjected to site-directed mutagenesis, and the mutant proteins were characterized for their ability to bind DNA and to physically and functionally interact with NKX2-5. The studies identify 5 highly conserved amino acids in the second zinc finger (N272, R283, Q274, K299) and its C-terminal extension (R319) that are critical for physical and functional interaction with the third alpha helix of NKX2-5 homeodomain. Integration of the experimental data with computational modeling suggests that the structural arrangement of the zinc finger-homeodomain resembles the architecture of the conserved DNA binding domain of nuclear receptors.

**Conclusions:**

The results provide novel insight into the structural basis for protein-protein interactions between two important classes of transcription factors. The model proposed will help to elucidate the molecular basis for disease causing mutations in GATA4 and NKX2-5 and may be relevant to other members of the GATA and NK classes of transcription factors.

## Introduction

Transcriptional networks orchestrate complex biologic processes. Such networks are commonly regulated through combinatorial interactions of transcription factors. GATA family transcriptional regulators and their co-factors control cell fate decisions in multiple tissues from worms to mammals [1–3]. A characteristic feature of all GATA factors is a structure with two adjacent zinc finger domains (Cys-X2-Cys-X17-Cys-X2-Cys), which mediates binding to a DNA consensus sequence (A/T)GATA(A/G). Of the six mammalian GATA transcription factors, GATA1, 2, and 3 are prominently expressed in hematopoietic cell lineages, whereas GATA4, 5, and 6 are expressed in mesoderm and endoderm derived tissues such as heart, liver, lung, gonad, and gut [1–3]. In the developing murine heart, GATA4 is one of the earliest-expressed transcription factors [4] and is indispensable for normal cardiac development [5, 6]. Importantly, GATA4 is necessary and its upregulation sufficient to induce cardiogenesis in embryonic stem cells [7] and to promote a cardiac cell fate from non-cardiogenic cells [8–10]. GATA4 has also been implicated in cardiac regeneration and repair [11, 12]. GATA4 is expressed also in the adult heart, acting as a key transcriptional regulator of numerous cardiac genes including those encoding atrial natriuretic peptide (ANP), B-type natriuretic peptide (BNP), α-myosin heavy chain (α-MHC), β-MHC, and many others [13, 14]. In the postnatal heart, GATA4 acts as a critical regulator of hormone response and mechanical stress as well as cardiomyocyte survival and myocardial remodeling [15–22].

GATA4 actions involve combinatorial interactions with a number of other nuclear proteins. Most of the interactions occur through the C-terminal zinc finger which also binds DNA and is highly conserved throughout the GATA family. Over 20 point mutations in the C-terminal zinc finger of GATA4 have been reported in patients with congenital heart disease. The mechanisms by which mutations cause cardiac defects remain largely undefined. Recently, the structure of the C-terminal zinc finger of hGATA4 has been resolved by NMR (RCSB-Protein Data Bank code: 2M9W) but the residues therein involved in contacting specific cofactors remain to be identified.

The cardiac specific homeobox protein NKX2-5, a member of the evolutionary conserved NK family of homeobox proteins and a critical GATA4 cofactor, is essential for heart development [14, 23]. Mutations in the NKX2-5 gene are associated with congenital heart disease (CHD) including septal defects and conduction system abnormalities [24, 25] but the mechanisms underlying pathogenesis have not been elucidated yet. GATA4 and NKX2-5 are co-expressed in cardiac progenitors where they are thought to be required to transduce cardiogenic, such as bone morphogenic protein (BMP), signals [26, 27]. GATA4 and NKX2-5 interact physically and synergistically activate several cardiac genes [14, 28–31] most notably those encoding the major secretory products of the heart: ANP and BNP. Here we combined experimental and molecular modeling data to achieve a better understanding of the structural basis for the GATA4-NKX2-5 interaction. A homology model was constructed and used to identify surface amino acids important for the interaction of GATA4 and NKX2-5. These residues were subjected to site-directed mutagenesis and the mutant proteins were characterized for their ability to bind DNA and to physically and functionally interact with NKX2-5 and with another GATA4 cofactor, Krüppel-like zinc finger KLF13 [32]. The results identify specific residues within the GATA4 second zinc finger domain and C-terminal extension that are differentially required for NKX2-5 interaction. The data shed important new insight into the structural basis for GATA4 action in the heart and provides a platform to analyse disease-causing mutations in GATA4 and NKX2-5.

## Materials and Methods

### Plasmids

The plasmid expressing the mouse GATA4 (pMT2-GATA4) and the empty pMT2 plasmid were gifts from D.B. Wilson (Department of Pediatrics, St. Louis Children's Hospital) [33] and the plasmid expressing the mouse NKX2-5 (pEF-FLAG-NKX2-5) was provided by R.P.Harvey (The Victor Chang Cardiac Research Institute, Darlinghurst, Australia) [34]. Mouse NKX2-5 cDNA sequence was cloned from pEF-plasmid to pMT2-plasmid to EcoRI-site using following oligos forward: ATATATGAATTCTCCAGATCTTTCGAAATCACCATGGACTAC, reverse: ATATATGAATTCCTTGCGTTACGCACTCACTTTAATGGGAAG. GATA4 mutations were generated into pMT2-GATA4 plasmid which contains mouse Gata4 cDNA [33] by using a QuikChange II Site-Directed Mutagenesis Kit (Stratagene) according to the manufacturer's protocol with the sense primers presented in Table 1. All constructs were fully sequenced to confirm the presence of the mutations and to ensure that no additional nucleotide changes had been introduced. The mutations are designated by their position in the mouse GATA4 protein sequence (GenBank Accession Number NP_032118). To create p3xHA luciferase reporter containing three NKX2-5 high affinity binding elements (NKE) (modified from the previous studies [34, 35]) two oligos with MluI and BglII restriction sites (sense; CGCGTCTCAAGTGGGTCTCAAGTGGAGCCTCAAGTGGA, antisense; AGAGTTCACCCAGAGTTCACCTCGGAGTTCACCTCTAG) with phosphorylated 5’ ends obtained from Oligomer (Helsinki, Finland) were annealed and ligated to pGL3 basic reporter vector containing rat albumin minimal promoter with TATA-box (-40 - +28), a kind gift from J. Hakkola (Department of Pharmacology and Toxicology, University of Oulu, Finland) [36]. Underlined sequences denote NKX2-5 high affinity binding site. Rat BNP minimal promoter containing luciferase reporter vector and minimal promoter together with -90 tandem GATA-site have been described previously [13]. Internal control vector pRL-TKd238 was a gift from J.F. Strauss III (University of Pennsylvania School of Medicine, Philadelphia) [37]. KLF13 expression vectors and the ANP-luciferase reporter have been previously described [32, 38].

**Table 1:** Primers used to create the GATA4 mutations.

Amino acid mutated	Primer 5' to 3' direction
V217Y	CAGAAGGCAGAGAGTGT**TAC**AATTGTGGGGCCATGTC
H234S	GAGATGGGACGGGA**AGC**TACCTGTGCAATGCC
R264A	CTGTCCGCTTCC**GCC**CGGGTAGGCCTC
S269C	GGGTAGGCCTC**TGC**TGTGCCAACTGCCAG
A271V	GCCTCTCCTGT**GTC**AACTGCCAGACTACC
N272S	CCTCTCCTGTGCC**AGT**TGCCAGACTACCACC
N272D	GTAGGCCTCTCCTGTGCC**GAT**TGCCAGACTACCAC
Q274H	CCAACTGC**CAT**ACTACCACCACCACGCTG
R283A	GCTGTGGCGT**GCT**AATGCCGAGGGTGAGC
R283Q	CGCTGTGGCGT**CAA**AATGCCGAGGGTGAGC
E288G	GCCGAGGGT**GGG**CCTGTATGTAATGCCTG
E288K	GCGTCGTAATGCCGAGGGT**AAG**CCTGTATG
M298Y	CGGCCTCTAC**TAC**AAGCTCCATGGGGTTCCCAGG
K299A	CGGCCTCTACATG**GCG**CTCCATGGGGTTC
R319C	CCAGAAAA**TGT**AAGCCCAAGAACCTGAATAAATCTAAGACGCC
R319S	CGGAAGGAGGGGATTCAAACCAGAAAA**AGC**AAGCCCAAGAAC
P321C	GGAGGGGATTCAAACCAGAAAACGGAAG**TGC**AAGAACC
S327A	GCCCAAGAACCTGAATAAA**GCT**AAGACGC

Highlighted nucleotides denote mutated codons.

### Cell culture and preparation of the samples

COS-1 cells were a kind gift from J. Hakkola (Department of Pharmacology and Toxicology, University of Oulu, Finland) and have originally been obtained from American Type Culture Collection (ATCC, Manassas, VA, USA). NIH3T3 (ATCC, Manassas, VA, USA) and COS-1 cells were cultured in Dulbecco's modified Eagle’s medium (Sigma/Gibco) containing 10% Fetal Bovine Serum (Gibco) and 1% Penicillin-Streptomycin (Sigma). For nuclear and total protein extractions COS-1 cells were seeded into 175 cm2 culture flasks and transfected 24 h later with 20 μg of the indicated expression vectors using FuGENE 6 (Roche) reagent. Transfection reagent was removed after 24 h and cells were collected 48 h post-transfection. Protein extracts were prepared as previously described [39, 40].

### Immunoprecipitation and immunoblotting

The relative expression levels of native and mutant GATA4 proteins were evaluated by western blot and used to adjust the required volume of cell extract from transfected cells in order to standardize the amount of GATA4 protein in each immunoprecipitation. The total protein concentration was kept equal between the samples by the addition of protein extract from untransfected cells as required. For each immunoprecipitation, 50 μg of protein from FLAG-NKX2-5 expressing cells, 30–84 μg of protein from GATA4 expressing cells and 0–54 μg of protein from non-transfected cells were used. Thirty microliters of agarose bound anti-FLAG M2 antibody (Sigma) was used per immunoprecipitation reaction followed by overnight incubation in lysis buffer (20 mM Tris, pH 7.5, 150 mM NaCl, 1 mM EDTA, 1 mM EGTA, 1% Triton X-100 and 2.5 mM sodium pyrophosphate) with protease and phosphatase inhibitors (20 μg/ml leupeptin, 2 μg/ml pepstatin, 20 μg/ml aprotinin, 1 mM phenylmethanesulfonyl fluoride (PMSF), 50 mM NaF, 6 μg/ml N-tosyl-L-phenylalaninyl-chloromethylketone (TPCK) and 6 μg/ml N-alpha-tosyl-L-lysinyl-chloromethylketone (TLCK)) at +4°C with gentle agitation. The beads were collected by quick spin and washed three times with lysis buffer. The immunoprecipitated proteins were eluted from the agarose beads by boiling the samples in SDS-loading buffer, resolved by SDS-PAGE as divided into duplicate, transferred onto Optitran BA-S 85 Reinforced nitrocellulose membrane (Schleicher & Schuell) and immunoblotted with anti-GATA4 polyclonal antibody (sc-9053, Santa Cruz Biotechnology) and anti-NKX2-5 polyclonal antibody (sc-8697, Santa Cruz Biotechnology). Horseradish peroxidase-conjugated anti-rabbit IgG or anti-goat IgG was used as secondary antibody. The immune complex was visualized by using an ECL detection kit (Amersham Pharmacia Biotech) followed by exposure to film (Hyperfilm ECL, Amersham Biosciences) or digitalization of chemiluminescence with Luminescent Image Analyzer LAS-3000 (Fujifilm). All immunoreactive bands were quantified using Quantity One software (Bio-Rad).

For the analysis of the results, the input GATA4 bands were normalized to input wtGATA4, i.e. control bands. Similarly, the NKX2-5 bands were normalized to control NKX2-5. First, the average intensity of the unspecific binding was subtracted from the intensity of the immunoprecipitated sample, which was then adjusted to input GATA4 level and normalized to control. These normalized values were finally adjusted to normalized NKX2-5 values. Each value from biological and technical replicates was treated separately and combined for statistical calculations.

### Electrophoretic mobility shift assay (EMSA)

Double-stranded oligonucleotides corresponding to the -90 GATA binding region of the BNP gene were used for the analysis of GATA4 DNA binding activity as previously described [39]. Probes used: rBNP -90 tandem GATA (sense 5'-TGTGTCT**GATA**AATCAGA**GATA**ACCCCACC-3'; antisense 5'- GGTGGGGTTATCTCTGATTTATCAGA-3'), rBNP GATAmut -91 (sense 5'- TGTGTCT**TGC** **A**AATCAGA**GATA**ACCCCACC-3'; antisense 5'-GGTGGGGTTATCTCTGATTTGCAAGA-3'), rBNP GATAmut -80 (sense 5'-TGTGTCT**GATA**AATCAGA**TGC** **A**ACCCCACC-3'; antisense 5'-GGTGGGGTTGCATCTGATTTATCAGA-3') and rBNP -91GATA (sense 5'-TGTGTCT**GATA**AATCAGAG-3'; antisense 5'-CTCTGATTTATCAGA-3'). GATA binding sites are indicated in bold and the mutated nucleotides are underlined. Anti-GATA4 polyclonal antibody (sc-1237X, Santa Cruz Biotechnology) was used for super shift assays. The bands were quantified using Quantity One software (Bio-Rad). For the analysis of the results each band was normalized to the average of the control bands. The normalized values were combined for statistical calculations.

### Luciferase reporter assay

In luciferase reporter assays COS-1 or NIH3T3 cells were co-transfected with 150 ng of Firefly luciferase reporter vector, 35 ng Renilla luciferase vector and 75 ng of expression plasmid pMT2-GATA4 per 1 cm2 well. In GATA4-NKX2-5 interaction studies with p3xHA the same amount of luciferase reporter vectors were used along with 75 ng of pMT2-NKX2-5 and 75 ng of pMT2-GATA4 plasmids. Particular GATA4 mutant proteins required adjustment in the transfected plasmid amount to achieve equal protein levels. Twenty-four hours after transfections cells were lysed with 1x Passive Lysis Buffer (E194A, Promega). The samples were processed with Dual-Luciferase Assay system according to manufacturer’s protocol (E1960, Promega) and measured with a luminometer (Luminoskan RS, Labsystems, Helsinki, Finland). NIH 3T3 cells were used to study the transcriptional activity of GATA4 mutants on the ANP promoter. Several doses of pMT2-GATA4 with or without varying doses of the other expression vectors were used to assess changes in transcriptional activities of GATA4 mutants over a wide range of DNA concentrations, which more accurately reflects transcriptional changes relative to the wt protein. Similarly, synergy with NKX2-5 was tested at different concentrations of GATA4 expression vectors to ensure the validity of the conclusions and to avoid artifacts due to the use of DNA concentrations outside the linear range. For the analysis of the results, the Firefly values were divided with the Renilla values and then normalized into control values. Each value from biological and technical replicates was treated separately and combined for statistical calculations.

### Molecular modeling

A homology model of GATA4 was constructed using the highly conserved zinc finger domains of GATA1 and GATA3 as template structures (PDB codes 1GNF for the N-terminal zinc finger and 3DFX for the C-terminal zinc finger of GATA4, sequence identities 84% and 76%, respectively). The model of the homeodomain of NKX2-5 was built by using the homeodomain of the related thyroid transcription factor 1 as a template (sequence identity 61%). Models built for the zinc fingers of GATA4 cover amino acids from 204 to 324 and for the homeodomain of NKX2-5 amino acids 146–198. The sequence alignments were constructed using PSI-BLAST and did not contain any insertions or deletions. The MOE software (Chemical Computing Group Inc, Canada) was used to construct the homology models of the protein domains. AMBER99 force field was applied as a source of atom parameters for all minimizations and scoring. Side chain data was assembled from an extensive rotamer library. Totally, 11 protein models were generated by the Boltzmann-weighted randomized modeling procedure (10 intermediate models and a refined final model). Each model was submitted to an electrostatics-enabled minimization run until Root Mean Square (RMS) gradient falls below 1. Protein models were scored by using the Generalized Born/Volume integral (GB/VI) methodology [41]. The best scoring intermediate model was further minimized until RMS falls below 0.5 and selected as a final model. Subsequently, the best scoring models were inspected and confirmed to have satisfactory stereochemical quality with Ramachandran plots [42].

### Statistical analysis

All the results are expressed as mean±SEM. To determine the statistical difference between two groups, Student’s t-test was used. In luciferase assays one group was considered as a control and all other groups were compared against it with Dunnett’s t-test using IBM SPSS Statistics software (Version 21.0. Armonk, NY: IBM Corp.). Differences at or above the 95% confidence level were considered statistically significant.

## Results

### Mapping of the key amino acids for GATA4-NKX2-5 physical interaction

**Fig 1:**
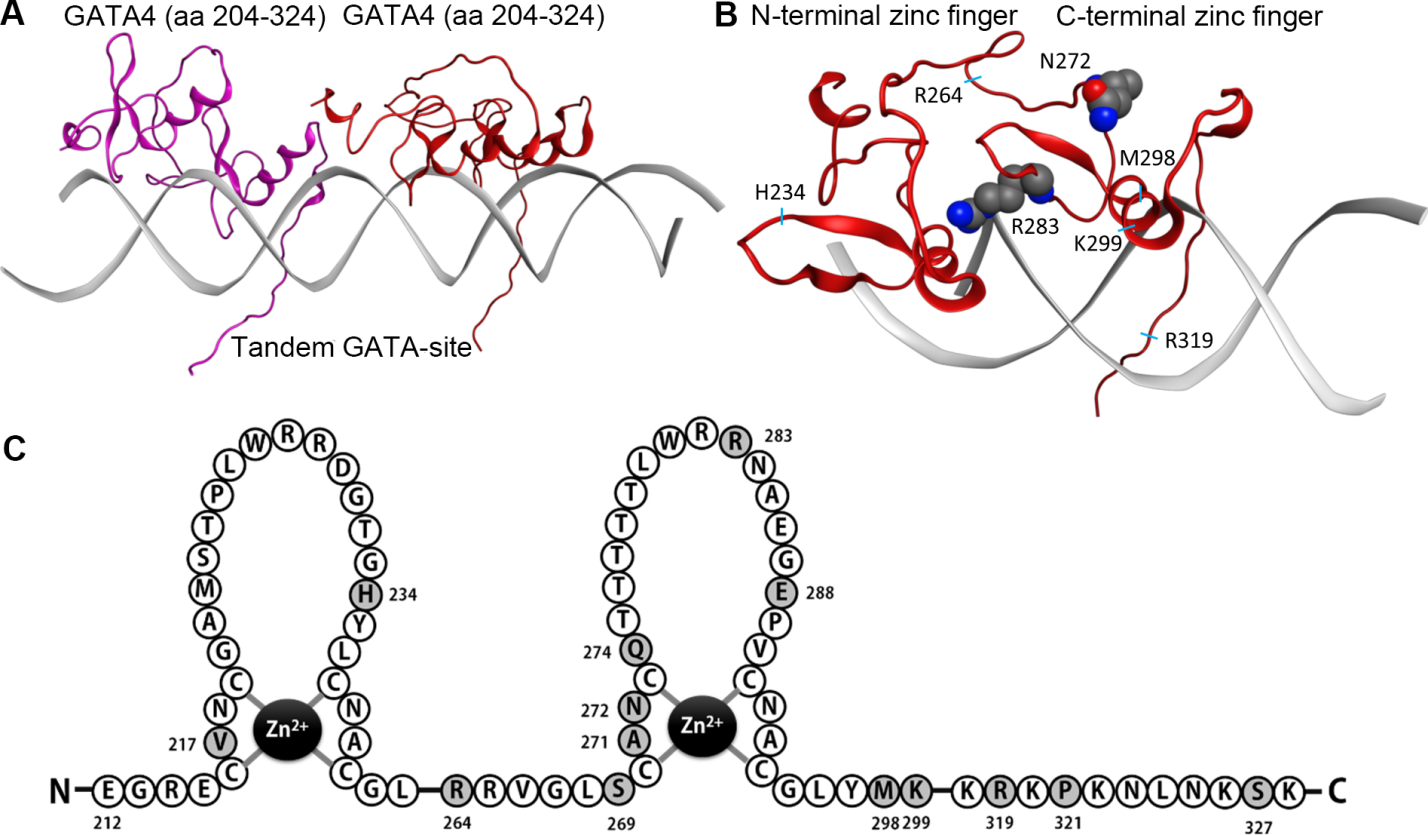
Homology models of transcription factor GATA4 bound to DNA. (A) Double GATA4 bound to a tandem GATA binding site. (B) C-terminal zinc finger of GATA4 bound to the major groove of DNA along with the N-terminal zinc finger bound to the minor groove. Amino acids N272 and R283 are highlighted as ball spheres and the location of amino acids H234, R264, M298, K299 and R319 are indicated. (C) Amino acid sequence of the mouse GATA4 zinc fingers. Darker shades indicate residues mutated in this study.

**Fig 2:**
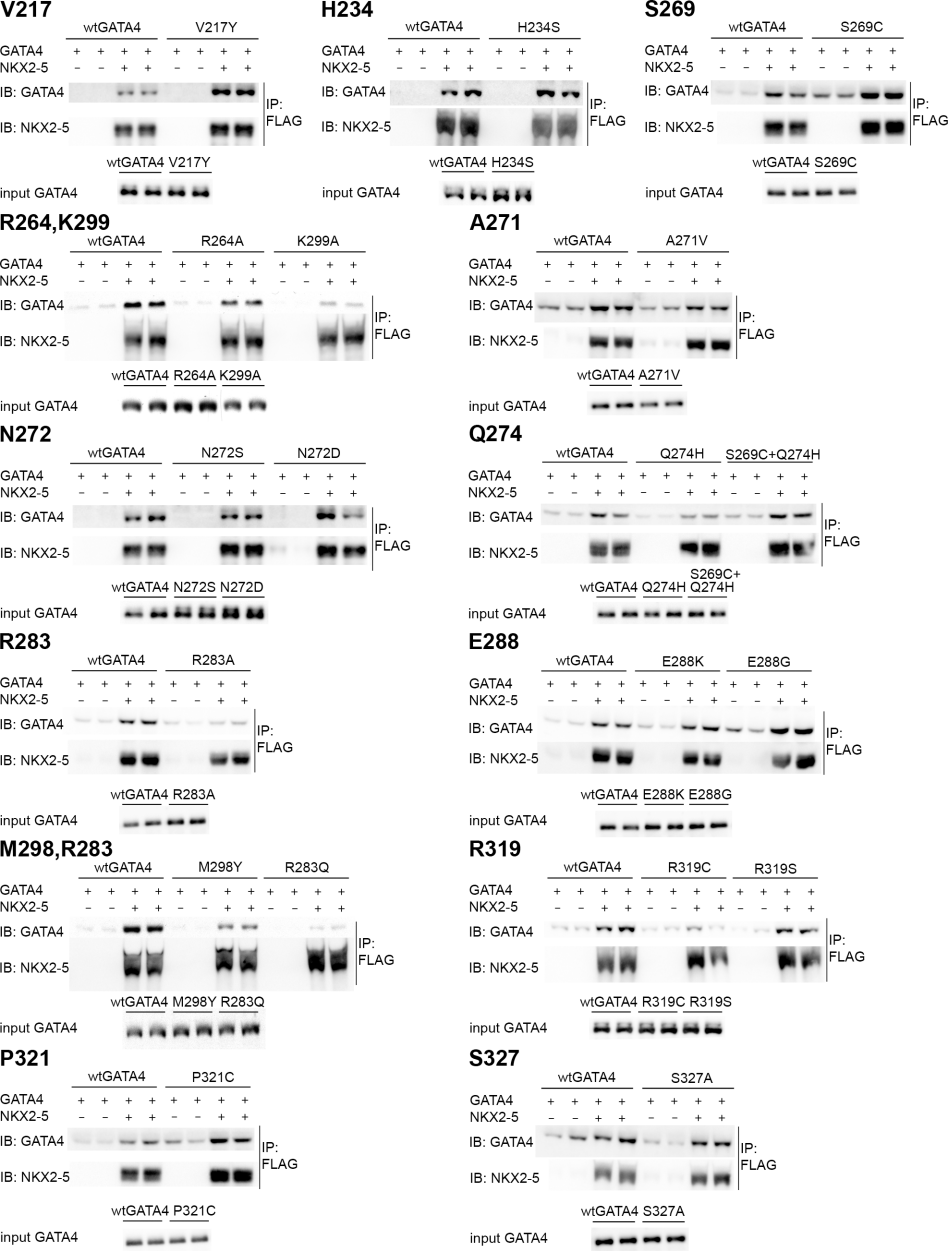
Representative Western blots following co-immunoprecipitation of GATA4 mutant proteins and NKX2-5-FLAG produced in COS-1 cells. For each GATA4 mutation immunoprecipitation was carried-out in two independent experiments with two technical replicates on western blot. The level of GATA4 protein in the input is shown below each co-ip blot. IP, immunoprecipitation; IB, immunoblotting

**Fig 3:**
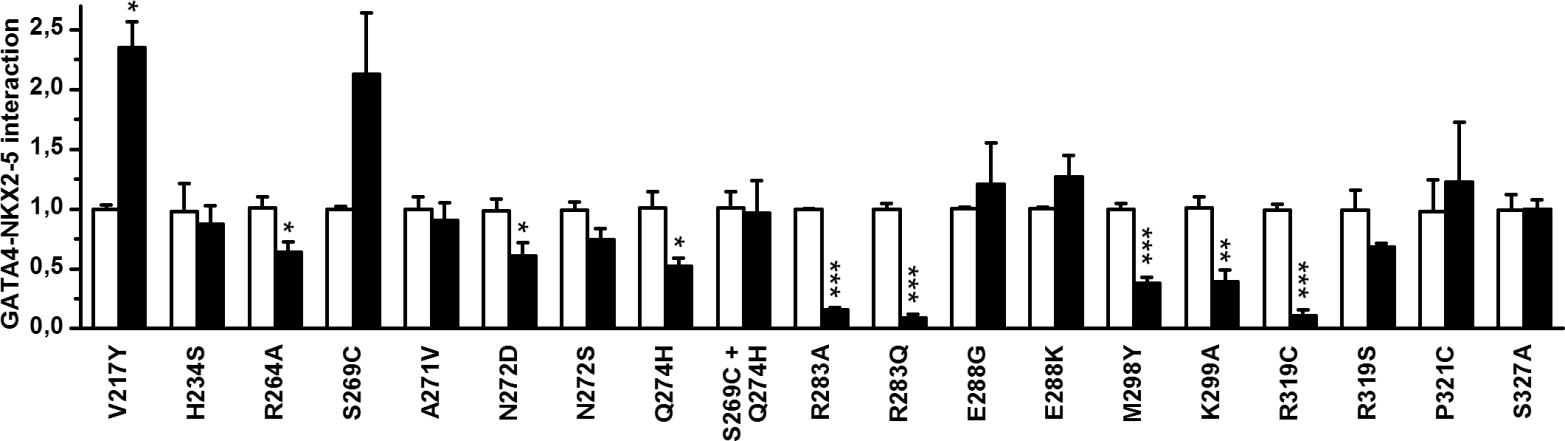
Effect of GATA4 mutations on interactions with NKX2-5. Quantitative analysis of GATA4 proteins binding to wtNKX2-5-FLAG. The effect of GATA4 mutations on GATA4-NKX2-5 interaction (black bar) is expressed as fold-changes compared to wtGATA4 control group (white bar). The raw data of the quantifications is available in S1 File. The results are presented as mean of two independent experiments ±SEM. * *P*<0.05, ** *P*<0.01 and *** *P*<0.001 vs. control group (Student's t-test).

The homology model of GATA4 was developed to design point mutations for identifying crucial amino acids for GATA4-NKX2-5 interaction (Fig 1A–1C). Preferably, the amino acids were selected to evenly cover the surface of the C-terminal zinc finger of GATA4 excluding the amino acids needed for DNA binding. Previous studies indicated that the second zinc finger and a C-terminal extension were required to contact NKX2-5 [14]. We therefore focused on these regions. Thirteen C-terminal zinc finger point mutations (R264A, S269C, A271V, N272D, N272S, Q274H, S269C+Q274H, R283A, R283Q, E288G, E288K, M298Y, K299A) and four C-terminal extension mutations (R319C, R319S, P321C, S327A) of GATA4 protein were generated. In addition, two mutations were produced in the N-terminal zinc finger (V217Y, H234S). The positions of all mutated residues are depicted in Fig 1C. Since all mutations were located outside the antibody recognition site, western blots were used to quantify the mutant protein levels produced in mammalian COS-1 cells. All mutant proteins are expected to localise to the nucleus like native GATA4 since single amino acid substitution in zinc finger area is not sufficient to inhibit the nuclear trafficking [43, 44]. Physical interactions with NKX2-5 were assessed in co-immunoprecipitation with N-terminal FLAG-NKX2-5 and analysed by western blots using GATA4 and NKX2-5 antibodies (Fig 2). The input samples confirmed the amount of GATA4 proteins used in the assays. Each mutation was studied in duplicate experiments with two technical replicates. Quantifications were performed on two reproducible blots and are presented in Fig 3 (raw data available in S1 File).

First, we analysed the effects of mutations in the GATA4 C-terminal zinc finger. Single amino acid mutations of E288K or E288G had no effect on interaction while R283A significantly decreased binding of GATA4 with NKX2-5 (84%, p<0.001). Substitution of arginine with the similarly sized but uncharged amino acid glutamine (R283Q) also significantly inhibited GATA4-NKX2-5 binding (91%, p<0.001) (Fig 3). Based on the homology model of GATA4, we created mutations R264A and K299A, which locate adjacent to and on the same surface but opposite direction to R283 (Fig 1B). Both mutations significantly decreased GATA4 binding to NKX2-5 (R264A, 36% p<0.05; K299A, 61%, p<0.01) (Fig 3). These results indicate that the three basic residues R264, R283 and K299 are important in contacting NKX2-5.

Next, we analysed the contribution of the amino acid M298, which is located in the helix. The side chain orientation of M298 is opposite to K299 (Fig 1B) and may provide a protein-protein interface. Mutation of this residue to tyrosine (M298Y), the amino acid found at this position in the hematopoietic GATA1-3 subclass, decreased GATA4 binding to NKX2-5 by 62% (p<0.001) (Fig 3). Mutation of N272 locating near M298 in the folded GATA4 protein (Fig 1B) is also found in humans (N273S) and causes heart malfunctions [45]. Interestingly, N272D also reduced GATA4-NKX2-5 binding albeit to a lesser extent (Fig 3). On the other hand, when the amino acid next to N272 in the loop area was mutated (A271V), no significant effect on the interaction between the two proteins was noted (Fig 3). By contrast, mutation of Q274 to histidine, the corresponding amino acid in GATA5 and GATA6 [1] decreased the interaction by 47% (p<0.05). Mutation of another residue, S269 to the corresponding GATA5 amino acid (S269C) had no significant effect (Fig 3). The double mutant protein (S269C+Q274H) which mimics GATA5 was as effective as wild type (wt) GATA4 in NKX2-5 interaction consistent with the ability of both wild type and mutant proteins to similarly synergize with NKX2-5.

In addition to the second zinc finger, GATA4 C-terminal extension (AA 303–390) is important for binding NKX2-5 [14]. C-terminal extension mutations R319C, R319S or P321C were created to mimic the amino acid combination observed in the protein model for NKX2-5 interface (Fig 1C). Here we intended to design an internal competing motif that could block NKX2-5 binding. Additionally, R319S was tailored to avoid possible formation of sulphur bridges in the experiment. A putative phosphorylation site S327 was also mutated (S327A), but this had no effect on NKX2-5 binding. R319C strongly decreased the GATA4-NKX2-5 interaction (89%, p<0.001) (Fig 3). On the other hand, R319S and P321C had no significant effects. Finally, as control, we produced two mutations in the N-terminal zinc finger. Amino acid V217 has been shown to be important for GATA4-FOG2 interaction [46]. Interestingly, V217Y mutation strongly increased (235%, p<0.05) GATA4-NKX2-5 binding (Fig 3), while mutation of H234S, located next to V217, had no effect (Fig 3). We conclude that of the amino acids tested, the significant structural determinants for GATA4 interaction with NKX2-5 are R264, N272, Q274, R283, M298, K299 and R319.

### Role of specific amino acids in GATA4 DNA binding

**Fig 4:**
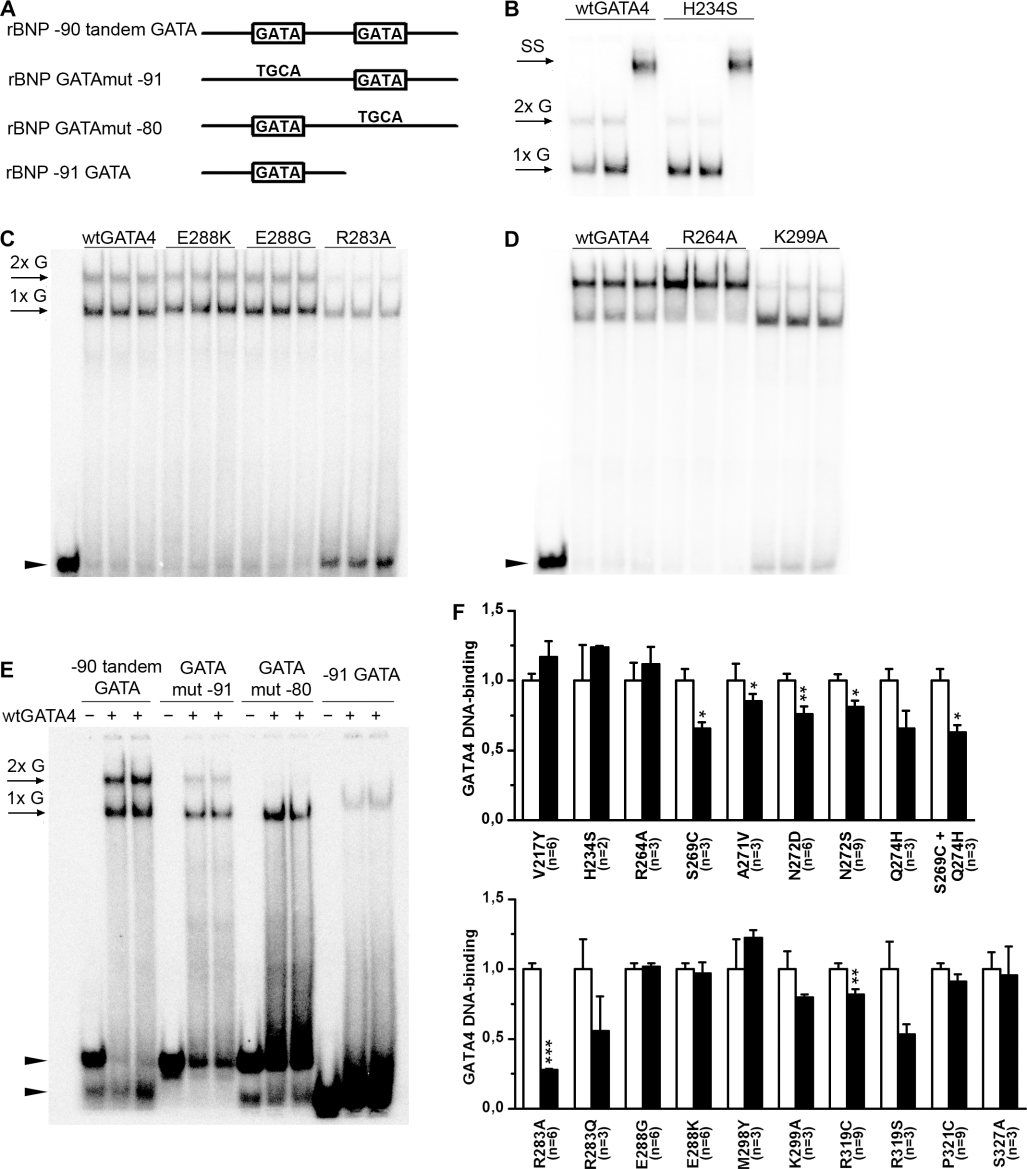
GATA4 binding to the tandem GATA site of the BNP promoter. (A) Schematic representation of the probes used in EMSA studies. (B) GATA4 binding to rBNP -90 tandem GATA site created two different bands. Supershift (SS) with 4 μg of GATA4 antibody C-20 confirms GATA4 presence in both bands. (C) The rBNP -90 tandem GATA site was used to study the effect of mutations on DNA binding. (D) R264A and K299A mutant proteins caused distinct binding pattern. (E) WtGATA4 binding to tandem GATA sites was studied with different mutated probes. 2xG = binding of two GATA4 molecules, 1xG = binding of one GATA4 molecule. Arrowheads denote free probes. (F) Effect of the GATA4 mutations (black bar) on DNA binding activity is expressed as fold-changes compared to wtGATA4 control group (white bar). Results (mean±SEM) are averages from two to nine independent reproducible EMSA samples using the -90 tandem GATA probe (n = 1–3). * *P*<0.05, ** *P*<0.01 and *** *P*<0.001 vs. control group (Student's t-test).

The amino acids studied above are on the surface of the native folded GATA4 protein and are not expected to alter the GATA4 zinc finger structure. The effects of the particular mutations on GATA4 DNA binding properties were studied by EMSA using the tandem GATA binding region of the BNP promoter as a probe (Fig 4A). GATA4 binding to this region creates two bands on EMSA as confirmed in supershift experiments with the anti-GATA4 antibody (Fig 4B). Each mutation was analysed in from two to nine independent experiments. As expected, most of the mutations did not influence on DNA binding (Fig 4C–4F *)*. The greatest effect was caused by the R283A mutation, which decreased DNA binding by 72% (p<0.001) (Fig 4C and 4F). Of note, mutations R264A and K299A showed consistently distinct binding patterns in EMSA (Fig 4D): R264A enhanced binding in the upper band and K299A in the lower band. However, when the intensities of both bands were added there was no significant effect in total DNA binding (Fig 4F). To explore if the two GATA elements were needed for GATA4 binding, DNA binding was tested with probes containing only one GATA site (Fig 4A and 4E). Mutation of the distal GATA site (GATA mut -91) reduced formation of the upper band, whereas mutation of the proximal GATA site (GATA mut -80) eliminated it; deletion of the entire proximal GATA site (-91 GATA), resulted in the formation of only one (the lower) band. These results suggest that the upper band represents binding of two GATA4 molecules to the tandem GATA elements as previously observed for GATA-1; [47] they also show that mutations in certain residues within the second zinc finger are needed for cooperative binding of GATA4 to DNA elements containing tandem GATA sites.

The model for GATA4 bound to DNA suggests that amino acids R264, R283 and K299 are located on the same side of the C-terminal zinc finger and are responsible for proper internal packing of the two zinc fingers. Upon DNA binding, the C-terminal zinc finger of GATA4 binds the major groove and the N-terminal zinc finger occupies the minor groove allowing interaction with the second GATA4 bound to the tandem GATA binding sites, located at a distance of 11 base pairs (Fig 1A). When either of the GATA elements was mutated, the binding of two GATA4 molecules was diminished. Interestingly, mutations of all these amino acids reduced the interaction with NKX2-5. In addition, K299A and R283A were associated with a unique gel shift pattern with the decreased ability to form dimers (Fig 4C and 4D). Together, these observations indicate that distinct intra- and intermolecular interactions are required to control GATA4 binding to various DNA regulatory elements.

### Functional effects of the GATA4 mutants

**Fig 5:**
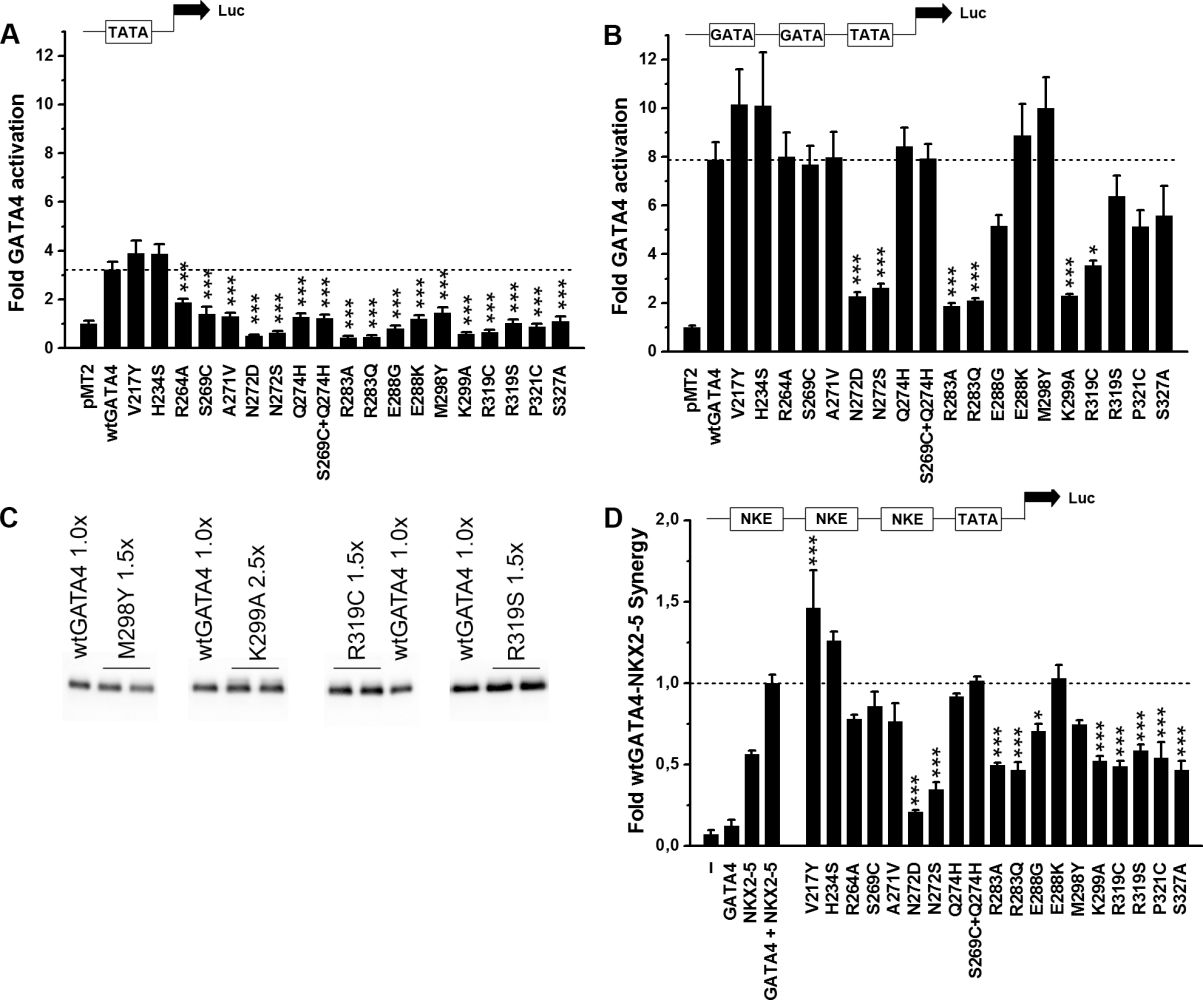
Effect of GATA4 mutations on transcriptional activity in COS-1 cells. (A) Results using the rat BNP minimal promoter and (B) the rat BNP promoter with the -90 tandem GATA sites (2XGATA). Results (mean±SEM) are expressed as fold-changes compared to pMT2 control group and are averages from three independent experiments with parallel samples (n = 3). (C) The mutant GATA4 proteins were expressed at the same level as the wtGATA4 protein. A representative western blot shows that 1.5-fold higher amount of the M298Y, R319C and R319S plasmids, and 2.5-fold higher of the K299A plasmid compared to the wtGATA4 were transfected to achieve equal protein levels. (D) A reporter construct containing three high affinity binding sites for NKX2-5 (NKE) was created to study transcriptional interaction between GATA4 and NKX2-5 proteins. The experiment was repeated two times with parallel samples (n = 2). The raw data of the luciferase assay is available in S2 File. Results (mean±SEM) are expressed as fold-changes compared to wtGATA4-NKX2-5 control group. * *P*<0.05, ** *P*<0.01 and *** *P*<0.001, vs. wtGATA4 in A and B and vs. wtGATA4-NKX2-5 in D (Dunnett’s t-test).

The effect of GATA4 mutations on transcriptional activity was studied using luciferase reporter assays (raw data available in S2 File). The minimal BNP promoter containing the first 60 bp including the proximal TATA-box (Fig 5A) as well as a reporter containing the minimal promoter and the -90 tandem GATA-sites (2XGATA, Fig 5B) were used. The expression level of the proteins was quantified by western blots and as shown in Fig 5C was similar for all GATA4 mutant proteins. Overall, when compared to the 3-fold activation by wtGATA4, all mutations tested, except the ones in the N-terminal zinc finger, showed decreased activation of the minimal promoter (p<0.001). The BNP promoter containing the -90 tandem GATA sites was activated 8-fold by wtGATA4 (Fig 5B). Only some of the GATA4 mutations affected transcriptional activity on this promoter. The strongest decrease in reporter activation was seen with mutants N272D, N272S, R283A, R283Q and K299A (67–76%, p<0.01).

GATA4 shows enhanced synergistic effect with NKX2-5 on promoters containing high affinity binding elements for NKX2-5 (NKE) [31, 48]. To evaluate the impact of mutations on functional interaction with NKX2-5, we first studied GATA4-NKX2-5 transcriptional synergy on an artificial construct containing 3xNKE. As shown in Fig 5D, wtGATA4 alone had no effect, while NKX2-5 activated the 3xNKE promoter alone and synergistically with GATA4. Strikingly, the strongest inhibitory effect on synergy was noted with GATA4 proteins containing N272 mutations (N272D 79% and N272S 65% reduction vs wtGATA4, p<0.001). Significant reduction (p<0.05) was also seen with mutations E288G, R283A, R283Q, K299A, R319C, R319S, P321C, and S327A. Finally, V217Y, which increased physical interaction with GATA4, also increased synergy with NKX2-5 in the luciferase reporter assays (146%, p<0.001) (Fig 5D).

### Effect of GATA4 mutations on the transcription of ANP promoter

**Fig 6:**
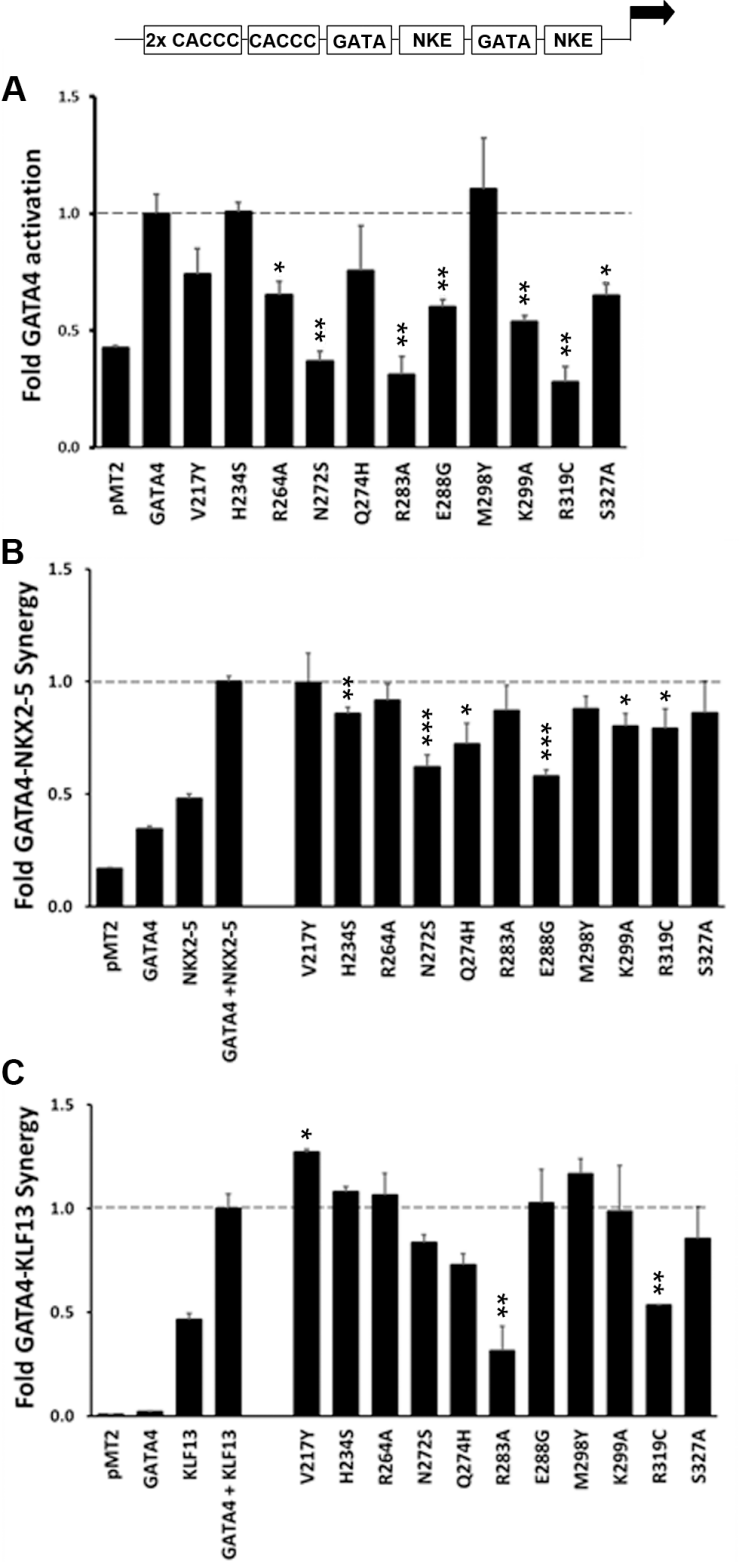
Effect of GATA4 mutations on ANP promoter activation in NIH3T3 cells. The -699bp ANP promoter was utilized to study the transcriptional activity of the GATA4 mutants on a validated GATA4 target gene. (A) Promoter activation by the indicated GATA4 proteins; activation by wtGATA4 is set at 1. (B) Effect of mutated GATA4 on synergy with NKX2-5 and (C) on synergy with KLF13. The data shown are from two independent experiments conducted in duplicate using five different DNA concentrations. Results (mean±SEM) are expressed as fold-changes compared to the control groups wtGATA4 in (A), wtGATA4-NKX2-5 in (B), and wtGATA4-KLF13 in (C). * *P*<0.05, ** *P*<0.01 and *** *P*<0.001 vs. control group with Student's t-test.

The effect of representative GATA4 mutations on the transcriptional activity of the ANP promoter was also studied using luciferase reporter assays. This promoter contains two high affinity GATA binding sites adjacent to NKE sites [14, 49, 50]. First, a dose response study was conducted to determine the profile of the GATA4 mutants on a natural GATA4 target promoter (data not shown). Several GATA4 mutations affected transcriptional activity: a significant decrease in GATA4 activation of the ANP reporter was observed with the point mutant K299A (52% p<0.01) and a complete loss of GATA4 transcriptional activation was observed with N272S, R283A, and R319C (p<0.01) (Fig 6A). This profile is quite similar to the one obtained with the longer BNP promoter (Fig 5B). Mutations R264A, E288G and S327A had a stronger effect on ANP promoter activation compared to that of BNP, likely reflecting the more complex protein-protein interactions on this promoter.

The same mutants were examined for their effect on cooperative activation of the ANP promoter with NKX2-5 (Fig 6B). Mutants N272S and E288G reduced the synergistic interaction with NKX2-5 most significantly (p<0.05). As with the 3xNKE reporter, K299A and R319C also decreased synergy.

### Effect of GATA4 mutations on interaction with other cofactors

To further define the specific amino acids for GATA4-NKX2-5 interaction we also examined the effect of GATA4 point mutations on the synergy with another GATA4 cofactor, KLF13 transcription factor. In contrast to NKX2-5, KLF13 contacts GATA4 via its N-terminal zinc finger [32]. As shown in Fig 6C, most GATA4 mutants did not affect synergy with KLF13 on ANP promoter. In fact, only two mutations significantly reduced synergy with KLF13, one in the second zinc finger (R283A) and the other in the basic domain (R319C). Interestingly, these mutations inhibited more effectively KLF13 synergy than NKX2-5 synergy.

### Proposed Model for GATA4-NKX2-5 interaction

**Fig 7:**
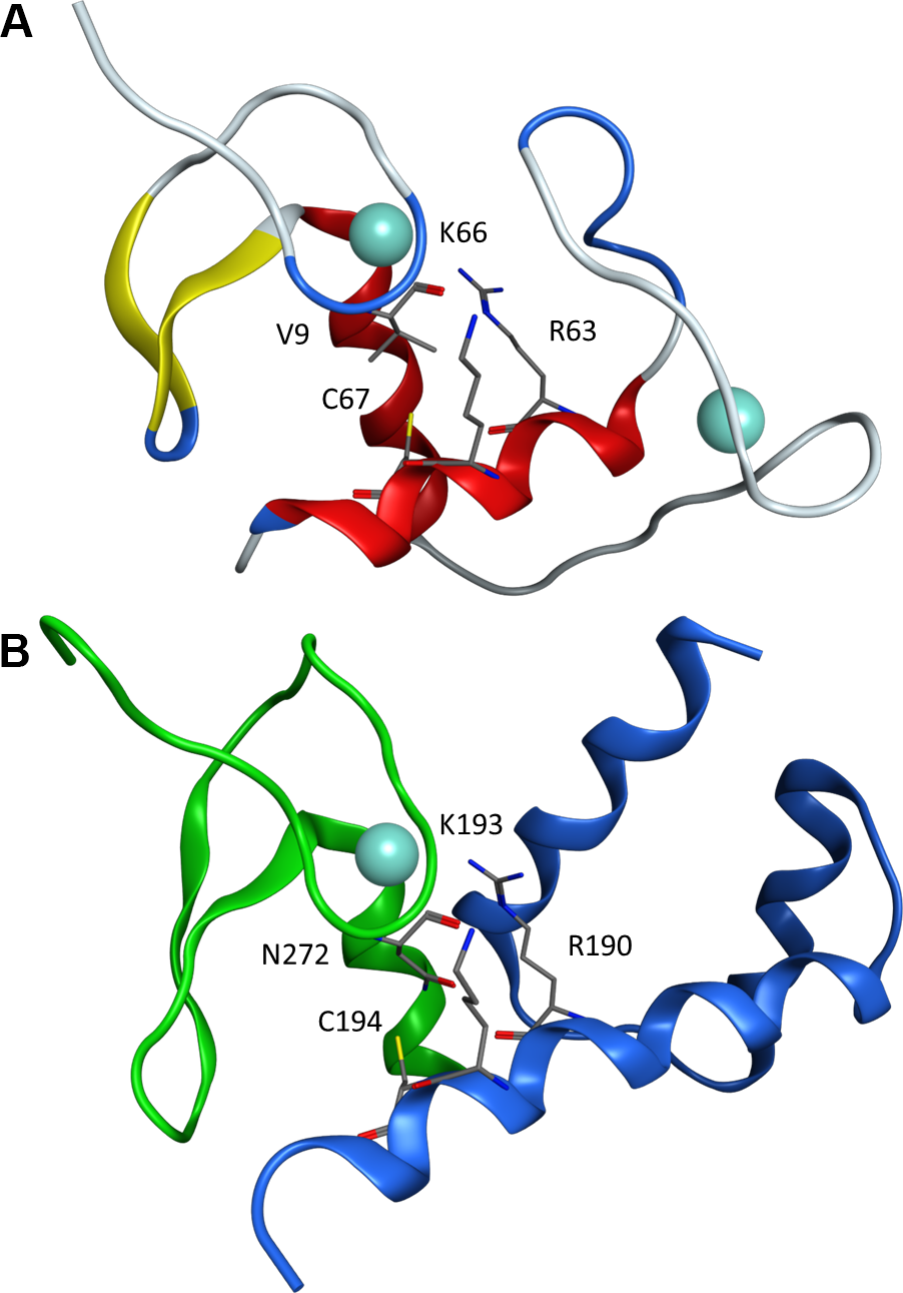
Nuclear receptor like structure of GATA4-NKX2-5 interaction. (A) DNA-binding domain of the estrogen receptor (Protein Data Bank: 1HCQ) showing R63, K66 and C67 mediating the interaction between the zinc fingers. (B) Homology model of the C-terminal zinc finger of GATA4 (green) and the homeodomain of NKX2-5 (blue) showing amino acids R190, K193 and C194 (S3 File). Structure similarity between GATA model and Estrogen receptor (1HCQ) is 1.81 RMSD (alpha-carbon). Due the sequence and structural fold differences between NKX2-5 and Estrogen receptor, RMSD values are not reported. Zinc atoms are represented as light blue spheres.

Nuclear receptors have two zinc fingers packed together with conserved amino acid residues mediating the interaction between the first and second zinc finger (Fig 7A). The C-terminal zinc finger of GATA4 shares the structural fold of other C4-type zinc fingers including the first zinc finger of nuclear receptors. On the other hand, helix III of the second zinc finger of these receptors contains a conserved helical structure mediating interaction between the two zinc fingers, such as estrogen receptor residues R63, K66 and C67 [51]. Detailed structural analysis revealed that helix III in the homeodomain of NKX2-5 has a nuclear receptor-like binding motif as well. Sterically accessible NKX2-5 amino acids R190, K193 and C194 are arranged similarly in a helical motif as the corresponding residues in the estrogen receptor (Fig 7B). Previously it has been shown that helix III of NKX2-5, especially K193, is required for association with the C-terminal zinc finger of GATA4 [52]. Remarkably, our model predicts that the interaction between GATA4 and NKX2-5 shares the same architecture seen for the two zinc fingers in the DNA binding domain of nuclear receptors (Fig 7B, S3 File).

## Discussion

GATA4 is a dosage-sensitive regulator of heart development and changes in GATA4 protein levels or activity may lead to congenital heart disease [53, 54]. Over 90 point mutations in GATA4 have been associated with human congenital heart disease with the majority found in the two zinc fingers and C-terminal domains. In most cases, the mechanisms of pathogenesis remain largely unidentified but it is assumed that mutations lead to haploinsufficiency. Indeed, in the few cases where the effect of the mutations have been tested, a decrease in GATA4 activity was noted [55–59]. However, several mutations did not have an effect on GATA4 transcriptional activity while others appear to be gain-of-function mutations [60]. The molecular basis underlying the changes in GATA4 activity caused by these single point mutations remains to be defined. Alterations in nuclear accumulation, DNA binding, or protein-protein interactions can have profound effects on GATA4 activity. In particular, differential effects on GATA4 interaction with its cofactors may help elaborate genotype-phenotype relations. So far, only a handful of GATA4 mutations have been shown to affect cofactor interactions. They include mutation in the N-terminal domain that alter Cyclin D2 interaction and that was identified in familial atrial fibrillation; [59] mutation in the first zinc finger that prevents FOG2 association and was found in testicular defects; [61] two mutations, one in the second zinc finger and the other in the adjacent nuclear localisation domain that prevent physical interaction with TBX5, found in familial tetralogy of fallot and in septal defects; [55, 58] and last, C271S in the N-terminal end of the second zinc finger that reduces synergy with NKX2-5 and was found in an autosomal dominant form of dilated cardiomyopathy [57]. A better understanding of the structure-function determinants of GATA4 is a prerequisite for elucidating the mechanisms by which mutations in GATA4 lead to defective heart formation and for developing genotype-phenotype relations.

As stated earlier, the role of GATA4 in the heart ranges from differentiation, survival and proliferation of cardiac progenitors to adaptive stress response, angiogenesis and myocardial repair of the post-natal heart. The diverse GATA4 functions are thought to involve differential interactions with cell-specific and signal-inducible cofactors. For example, interaction with the calcium-calcineurin inducible transcription factor NFAT may underlie the hypertrophic response [62] whereas cooperativity with growth factor responsive proteins such as Smad, STAT (signal transducers and activators of transcription) and Jun/Fos may be required for proliferative growth and for hormonal response [63, 64]. On the other hand, GATA4 is known to activate cardiac gene transcription synergistically with several classes of cardiac-specific transcription factors and while the exact role of these interactions is not fully elucidated, their evolutionary conservation, together with genetic and biochemical evidence point to important functions in heart development.

In this study, we conducted a mutagenic analysis of the second zinc finger domain aimed at elucidating the structural basis for GATA4-NKX2-5 interaction. The results summarized in Table 2 identify specific residues within this domain and its C-terminal extension that are involved in physical and functional interaction with NKX2-5. Integration of the experimental data with computational modelling suggests that the topology of the GATA4-NKX2-5 interaction is reminiscent of that observed between the two zinc fingers of nuclear receptors. The unexpected finding that GATA4-NKX2-5 interaction resembles that of nuclear receptors may have far-reaching implications for understanding the molecular consequences of pathogenic GATA4 and NKX2-5 mutations. Given the wealth of information on nuclear receptor structure, regulation and cofactors, the structural similarity unravelled from this work may assist studies aimed at clarifying the regulation and molecular components of GATA4-NKX2-5 transcription complexes.

**Table 2:** Summary of GATA4 structure function.

GATA4 mutation	DNA binding	Promoter activation	NKX2-5 binding	Synergy with NKX2-5 on 3xNKE	Synergy with NKX2-5 on ANP	Synergy with KLF13 on ANP
WT	Normal	Normal	Normal	Normal	Normal	Normal
V217Y	Normal	Normal	↑	↑	Normal	↑
H234S	Normal	Normal	Normal	Normal	↓	Normal
R264A	Altered	Normal	↓	Normal	Normal	Normal
S269C	↓	Normal	Normal	Normal	ND	ND
A271V	↓	Normal	Normal	Normal	ND	ND
N272D	↓	↓	↓	↓	ND	ND
N272S	↓	↓	Normal	↓	↓	Normal
Q274H	Normal	Normal	↓	Normal	↓	Normal
S269C+Q274H	↓	Normal	Normal	Normal	ND	ND
R283A	↓	↓	↓	↓	Normal	↓
R283Q	Normal	↓	↓	↓	ND	ND
E288G	Normal	Normal	Normal	↓	↓	Normal
E288K	Normal	Normal	Normal	Normal	ND	ND
M298Y	Normal	Normal	↓	Normal	Normal	Normal
K299A	Altered	↓	↓	↓	↓	Normal
R319C	↓	↓	↓	↓	↓	↓
R319S	Normal	Normal	↓	↓	ND	ND
P321C	Normal	Normal	Normal	↓	ND	ND
S327A	Normal	Normal	Normal	↓	Normal	Normal

↑ = increased vs. wt, ↓ = decreased vs. wt, ND = not determined

Earlier work has shown that the second zinc finger of GATA4 contacts NKX2-5 homeodomain and suggested that this interaction induces a conformational change that unmasks NKX2-5 activation domains [14]. This protein-protein interaction requires an intact zinc finger structure but was not strictly dependent on GATA4 binding since NKEs were sufficient to mediate synergy [48]. For NKX2-5, the C-terminus of the homeodomain which harbours the α3 helix and a short C-terminal extension of the homeodomain were needed to contact GATA4 [14, 50]. Based on our mutational studies and molecular modeling of interactions between GATA4 and NKX2-5, the GATA4 mutations created can be classified into different hot spots. In general, hot spots are a small subset of contact residues contributing significantly to the binding free energy and therefore are drivers for the protein-protein interactions [65]. Even the one amino acid mutation at the hot spot region has an influence to the interaction, and in addition to this, specific amino acid change play a substantial role leading to different surface properties (charge, size, hydrophobicity). First, mutations of the GATA4 residues R264, R283 and K299 resulted in loss of protein-protein interaction. Moreover, DNA binding experiments showed that these mutations create a distinct binding pattern and in particular affect cooperative binding over tandem GATA elements. The molecular model for the interaction between GATA4 and DNA suggests that C- and N-terminal domains are packed together and their orientation towards each other either activates or restricts binding of a second GATA4 protein to the promoter (Fig 1A). A similar intramolecular binding mode on palindromic GATA sites has been observed with GATA1 [47]. We assume that if R264 is mutated, N- and C-terminal zinc fingers are packed together and allow two GATA4 proteins to better bind this sequence. In contrast, K299A, adopting a more open conformation, allows only one GATA4 protein to be bound. The results of transcriptional assays are in agreement with this hypothesis. R264A is able to activate the promoter containing a tandem GATA-site similarly to wtGATA4, in contrast to K299A, which was unable to bind this sequence properly and to activate transcription. These results and their interpretation are consistent with the work of Philips et al. [43], who showed that mutation of R283 and K299 reduces DNA binding to a monomeric GATA element. Alterations in the packing of C- and N-terminal zinc fingers may thus be the cause for the strongly reduced GATA4-NKX2-5 physical interaction detected with these mutants.

Furthermore our studies demonstrate that N272 is a crucial amino acid for GATA4-NKX2-5 interaction, as shown by the strong inhibition of transcriptional synergy with NKX2-5 but not with KLF13. N272 is situated in close contact (approx. 5 Å) to M298. Since both mutations decreased the protein-protein interaction at least moderately and are located on the opposite side of the DNA binding surface of GATA4 (Fig 1B), we assume that this could be the potential binding site for NKX2-5. The effect of the N272 mutation was not clear-cut in immunoprecipitation assays. On the other hand, the relevance of this residue was more pronounced in our reporter assays, in which N272D and N272S were clearly unable to activate any of the promoters tested. This might be a consequence of small spatial or structural change in the physical assembly of the interacting proteins as a protein complex leading to unpredictable and strong effects in function. N272 is located specifically at the site that is analogous to the binding surface needed for internal packing of zinc fingers in nuclear receptors; M298 and N272 form a site on the protein surface that could harbor the cysteine containing sequence of α-helix III in NKX2-5.

Lastly, from the nuclear receptor analogy, we deduced that the binding interface should contain at least a successive cysteine and lysine in a helix structure to enable binding. Mutations R319C and R319S were designed to inhibit the protein-protein interaction by creating an internal protein motif, which might compete with NKX2-5 by occupying its binding site. Indeed, mutations at R319 (to cysteine or serine) showed decreased synergistic activity with NKX2-5 that was most severe on the NKE-dependent promoter. The finding that residues in the GATA4 basic domain are involved in modulating NKX2-5 interaction is consistent with our initial report on GATA4-NKX2-5 cooperativity [14]. In summary, we find that GATA4 amino acids N272, R283, K299, R319 are essential for interaction with NKX2-5 consistent with the structural model presented. These results may help explain the basis for several GATA4 mutations associated with congenital heart disease, including N273S, R283H, N285L and K319E [45, 66].

From the NKX2-5 perspective, half of the reported 40 heterozygous human mutations reside in the homeodomain [24]. The mechanisms by which these mutations cause disease, most notably atrial septal defects and AV block, remain to be elucidated. The model proposed here predicts that NKX2-5 amino acids R190, K193 and C194 would be involved in contacting GATA4. Consistent with this, analysis of the R190H and neighboring G187H human mutations showed that these changes abrogate physical interaction between NKX2-5 and GATA4 but do not affect NKX2-5-TBX5 interaction while Y191C and N188K did not disrupt the interaction between GATA4 and NKX2-5 [67]. These data further support our proposed model which may provide a helpful basis for understanding disease-causing mutations in these two critical cardiac regulators.

## Supporting Information

S1 File Immunoprecipitation western blot quantification data. (XLSX)

S2 File Luciferase assay data. (XLSX)

S3 File Homology model for C-terminal zinc finger of GATA4 and homeodomain of NKX2-5. (PDB)
